# Plasma Chemokine Levels Are Associated with the Presence and Extent of Angiographic Coronary Collaterals in Chronic Ischemic Heart Disease

**DOI:** 10.1371/journal.pone.0021174

**Published:** 2011-06-22

**Authors:** Ellen C. Keeley, J. Randall Moorman, Ling Liu, Lawrence W. Gimple, Lewis C. Lipson, Michael Ragosta, Angela M. Taylor, Douglas E. Lake, Marie D. Burdick, Borna Mehrad, Robert M. Strieter

**Affiliations:** 1 Division of Cardiology, University of Virginia, Charlottesville, Virginia, United States of America; 2 Department of Medicine, University of Virginia, Charlottesville, Virginia, United States of America; 3 Division of Pulmonary and Critical Care Medicine, University of Virginia, Charlottesville, Virginia, United States of America; 4 Department of Statistics, University of Virginia, Charlottesville, Virginia, United States of America; Leiden University Medical Center, The Netherlands

## Abstract

**Background:**

In patients with chronic ischemic heart disease (IHD), the presence and extent of spontaneously visible coronary collaterals are powerful determinants of clinical outcome. There is marked heterogeneity in the recruitment of coronary collaterals amongst patients with similar degrees of coronary artery stenoses, but the biological basis of this heterogeneity is not known. Chemokines are potent mediators of vascular remodeling in diverse biological settings. Their role in coronary collateralization has not been investigated. We sought to determine whether plasma levels of angiogenic and angiostatic chemokines are associated with of the presence and extent of coronary collaterals in patients with chronic IHD.

**Methodology/Principal Findings:**

We measured plasma concentrations of angiogenic and angiostatic chemokine ligands in 156 consecutive subjects undergoing coronary angiography with at least one ≥90% coronary stenosis and determined the presence and extent of spontaneously visible coronary collaterals using the Rentrop scoring system. Eighty-eight subjects (56%) had evidence of coronary collaterals. In a multivariable regression model, the concentration of the angiogenic ligands CXCL5, CXCL8 and CXCL12, hyperlipidemia, and an occluded artery were associated with the presence of collaterals; conversely, the concentration of the angiostatic ligand CXCL11, interferon-γ, hypertension and diabetes were associated with the absence of collaterals (ROC area 0.91). When analyzed according to extent of collateralization, higher Rentrop scores were significantly associated with increased concentration of the angiogenic ligand CXCL1 (p<0.0001), and decreased concentrations of angiostatic ligands CXCL9 (p<0.0001), CXCL10 (p = 0.002), and CXCL11 (p = 0.0002), and interferon-γ (p = 0.0004).

**Conclusions/Significance:**

Plasma chemokine concentrations are associated with the presence and extent of spontaneously visible coronary artery collaterals and may be mechanistically involved in their recruitment.

## Introduction

Chronic ischemic heart disease (IHD), the most common clinical manifestation of coronary artery disease, results in progressive myocardial ischemia due to gradual narrowing of the coronary arterial lumina and is manifested clinically as medically refractory angina, ischemic cardiomyopathy, congestive heart failure, and cardiac arrhythmias [1]. A major compensatory mechanism in patients with chronic IHD is the recruitment of coronary collaterals, a form of vascular remodeling that can be quantified angiographically. Presence of spontaneously visible coronary collaterals is associated with better outcomes in a broad spectrum of patients with varying degrees of IHD burden [2], including patients with acute myocardial infarction [3], [4], [5], [6], [7], [8], and patients with chronic IHD undergoing percutaneous [9], [10] and surgical [11], [12], [13] coronary revascularization. Recruitable coronary collaterals have also been assessed in patients with chronic IHD and are similarly associated with improved clinical outcomes [14]. The assessment of recruitable collaterals in the absence of chronic coronary occlusion, however, requires balloon occlusion of the collateral-receiving artery with simultaneous angiography of the collateral-supplying artery [15]. Thus, the physiological relevance of recruitable collaterals is limited to the context of complete coronary obstructions, whereas the presence of spontaneous collaterals can be seen in situations where lesions are flow-limiting but not necessarily completely occlusive.

Patients with chronic IHD and similar degrees of coronary artery stenosis exhibit marked variability in the presence of spontaneously visible collaterals, but the biological basis of this heterogeneity is not known [16]. Studies of the mechanisms underlying the recruitment of coronary collaterals have, in large part, concentrated on the potential contribution of growth factors including vascular endothelial growth factor (VEGF) and basic fibroblast growth factor (bFGF) with inconsistent results [17], [18], [19], [20], [21]. Thus, the mechanisms that contribute to the successful recruitment of coronary collaterals remain obscure.

Chemokines are a superfamily of structurally homologous cytokines that were originally described for their role in mediating leukocyte recruitment, but were subsequently found to be important regulators of vascular remodeling in diverse biological settings [22]. Chemokines are structurally defined by four conserved cysteine residues at their amino terminus and are classified into CC, CXC, C, and CX_3_C families based on the sequence of aminoacids in relation to the first 2 cysteine residues. The CXC ligands are further subdivided on the basis of presence or absence of a glutamic acid-leucine-arginine sequence (Glu-Leu-Arg or ‘ELR’ motif) immediately adjacent to the CXC motif [23]. The presence of this ELR motif is functionally important, since the ELR-containing CXC chemokine ligands (CXCL1, CXCL2, CXCL3, CXCL5, CXCL6, and CXCL8) are potent promoters of vascular remodeling, whereas a subset of the non-ELR CXC chemokine ligands (CXCL4, CXCL9, CXCL10, CXCL11) are potent inhibitors of angiogenesis [22], [23]. The contribution of the CXC chemokines to vascular remodeling of the myocardium in chronic IHD has not been studied in detail. The aim of this study was to test the hypothesis that in patients with chronic IHD, a profile of circulating levels of CXC chemokines is associated with the presence and extent of spontaneously visible coronary collaterals. We found that chemokines play an important role in both the presence and extent of coronary collaterals.

## Methods

### Objectives

To test the hypothesis that in patients with chronic IHD, a profile of circulating levels of CXC chemokines is associated with the presence and extent of spontaneously visible coronary collaterals.

### Participants

We prospectively collected demographic and angiographic data from consecutive patients referred for coronary angiography at the University of Virginia from October 2007 to August 2008. All patients undergoing coronary angiography who were >21 years old and able to provide informed consent were eligible for enrollment. Exclusion criteria were: (1) patients with acute coronary syndromes as defined by unstable or recent progression or acceleration of symptoms and/or elevated blood troponin level; (2) hematocrit <30; (3) active inflammatory, infectious, or malignant disease; (4) expected survival less than one year; and (5) immunosuppressive therapy.

### Description of Procedures

Following vascular access via the femoral, brachial or radial artery and prior to coronary angiography or heparin administration, a 30ml peripheral blood sample was drawn from the side-arm of the sheath, anticoagulated with sodium EDTA, immediately placed on ice, and processed within 30 minutes of retrieval. Platelet-free plasma was aliquoted and frozen at −80°C for subsequent measurement of: CXCL1, CXCL3, CXCL5, CXCL8, CXCL9, CXCL10, CXCL11, CXCL12, CCL2, VEGF and bFGF, and interferon-gamma (IFN-γ) by multiplex immunoassay using the manufacturer's instruction (Luminex, Bio-Rad, Bio-plex 200 system, Hercules, California; Procarta Cytokine Assay kit, Panomics, Inc., Fremont, California).

Selective coronary angiography was performed in multiple orthogonal views using standard techniques. Each angiogram was initially read by the interventional cardiologist performing the procedure (L.W.G., E.C.K., L.C.L., M.R., or A.M.T.), and the presence, severity, and extent of coronary artery disease was determined and entered into the cardiac catheterization laboratory computerized database. Angiograms reported to have at least one major epicardial artery with a severe stenosis (defined as ≥90% on visual estimation) were re-analyzed separately by 2 independent investigators (E.C.K. and L.L.) blinded to the clinical data and cytokine levels for the presence and extent of coronary collaterals. Since it has been previously shown that a severe flow-limiting coronary artery lesion (defined as a diameter stenosis of ≥90%) is a prerequisite for spontaneous collateral recruitment [24], only patients in whom the angiogram documented at least one lesion ≥90% were included. The presence and extent of collaterals (if present) was determined according to the Rentrop score [25]. If there was no evidence of a coronary collateral on the angiogram, it was documented as Rentrop = 0; if a collateral was present, it was further graded according to the Rentrop score as follows: Rentrop 1 =  filling of side branches of the artery without visualization of epicardial segments; Rentrop 2 =  partial filling of an epicardial artery; and Rentrop 3 =  complete filling of an epicardial artery. Disparities in Rentrop scores in angiograms from 4 patients were refereed by a third blinded investigator (L.C.L.) and data reflecting agreement from 2 of the 3 readers was used for analyses. If the patient had more than one collateral, the collateral with the highest Rentrop score was recorded and used for statistical analyses. The reproducibility of the Rentrop score has been previously described [26].

### Ethics

This study was approved by the institutional review board of the University of Virginia and all patients provided written informed consent.

### Statistical methods

The Fisher's Exact test was used to compare categorical values between patients with and without collaterals and the Wilcoxon rank-sum test was used for continuous variables. Difference of variables by Rentrop score were quantified using non-parametric ANOVA analysis (Kruskal-Wallis test). All continuous variables are reported as medians with interquartile ranges (IQR). A logistic regression model using a stepwise backward-selection technique was used to generate a multivariable model to determine the factors associated with the presence or absence of collaterals. The likelihood ratio test was used to test the significance of the variables. Candidate variables included age, extent of coronary artery disease, hypertension, diabetes, tobacco use, family history of coronary artery disease, hyperlipidemia, history of angina, history of peripheral vascular disease, history of congestive heart failure, history of arrhythmias, history of stroke, prior myocardial infarction, prior coronary artery bypass surgery, prior percutaneous coronary intervention, presence of an occluded artery and HMG Co-A reductase inhibitor therapy. A stepwise backward selection was performed using a threshold of *p*<0.10. All analyses were performed with SAS 9.1 (SAS Institute, Cary, NC) or MATLAB 7.9 (Natick, MA). A two-sided *p* value of <0.05 was considered statistically significant.

## Results

We enrolled 275 consecutive patients of whom 156 (57%) had a stenosis of ≥90% in at least one major epicardial coronary artery. Clinical characteristics of the 156 patients are shown in Table 1. Of the 156 patients, 88 (56%) had angiographic evidence of collateralization. There were no significant differences in baseline demographics (including race, data not shown), cardiac risk factors, medical co-morbidities, and chronic medications between patients with and without collaterals. Patients with collaterals had a higher mean number of diseased vessels (2.4+/−1.2 vs 1.9+/−1.0, *p* = 0.009), and were more likely to have at least one occluded coronary artery (85% vs 28%, *p*<0.0001), while patients without collaterals were more likely to have disease of a single coronary artery (43% vs 25%, *p* = 0.025) (Table 1).

**Table 1:** Clinical characteristics of patients with ≥90% stenosis of at least one coronary artery.

	with collaterals (n = 88)		no collaterals (n = 68)		*p* value
Demographic data					
Men	66 (75%)		50 (74%)		0.855
Age (yrs, mean +/− SD)	62±11		62±12		0.916
Cardiac risk factors					
Diabetes	29	(33%)	31	(46%)	0.135
Hypertension	71	(81%)	62	(91%)	0.073
Hyperlipidemia	81	(92%)	58	(85%)	0.203
Current tobacco use	30	(34%)	25	(37%)	0.739
Peripheral vascular disease	27	(31%)	19	(28%)	0.727
Family history of coronary artery disease	43	(49%)	30	(44%)	0.628
Medical history					
Prior stroke	8	(9%)	10	(15%)	0.318
Prior angina pectoris	50	(57%)	41	(60%)	0.744
Congestive heart failure	9	(10%)	6	(9%)	1.000
Prior myocardial infarction	40	(45%)	24	(35%)	0.251
History of cardiac arrhythmias	8	(9%)	7	(10%)	0.792
Prior coronary artery bypass surgery	23	(26%)	15	(22%)	0.579
Prior percutaneous coronary intervention	27	(31%)	26	(38%)	0.310
Chronic medications					
Beta-blocker	64	(73%)	53	(78%)	0.576
Angiotensin-converting enzyme inhibitor	44	(50%)	37	(54%)	0.746
Aspirin	79	(90%)	63	(93%)	1.000
Insulin	12	(14%)	17	(25%)	0.098
Oral hypoglycemic	11	(13%)	7	(10%)	0.802
Calcium channel-blocker	17	(19%)	9	(13%)	0.288
Clopidogrel	20	(23%)	15	(22%)	0.849
HMG coA-reductase inhibitor	68	(77%)	52	(76%)	0.693
Extent of coronary artery disease					
Number of diseased vessels	2.4+/−1.2		1.9+/−1.0		0.009
1-vessel disease	22	(25%)	29	(43%)	0.025
2-vessel disease	27	(31%)	19	(28%)	0.727
3-vessel disease	26	(29%)	17	(25%)	0.590
>3 vessel disease	13	(15%)	3	(4%)	0.060
Presence of any occluded artery	75	(85%)	19	(28%)	<0.0001

### Determinants of the presence of collaterals

In order to determine whether specific angiogenic and angiostatic factors and clinical characteristics were associated with either the *presence* or *absence* of collaterals we first performed a univariate analysis (Table 2). Subsequently, we performed a multivariate analysis (Table 3) that included all the cytokines and clinical characteristics listed in Table 2. In this best-fit model (ROC area 0.91; sensitivity 80%, specificity 88% at an optimal cutpoint), the factors associated with the presence of collaterals were the chemokine profile consisting of CXCL5 (*p* = 0.018), CXCL8 (p = 0.031), and CXCL12 (*p* = 0.033), and the clinical factors of hyperlipidemia (*p* = 0.047), and the presence of an occluded artery (*p*<0.0001). Factors associated with the absence of collaterals were the cytokine profile consisting of CXCL11 (*p* = 0.002) and IFN-γ (p = 0.021), and the clinical factors of hypertension (*p* = 0.007), and diabetes (*p* = 0.004). The remaining cytokines, including VEGF and bFGF, were not associated with the presence or absence of collaterals in the best-fit model.

**Table 2:** Univariate predictors of the presence of coronary artery collaterals.

Variable	Estimate (x10^4^)	*p* value
Angiogenic and angiostatic factors		
* * Angiogenic		
CXCL1	+0.022	0.428
CXCL3	+2.895	0.056
CXCL5	+0.406	0.041
CXCL8	+1.154	0.277
CXCL12	+0.579	0.169
CCL2	+5.445	0.246
VEGF*	−0.385	0.604
bFGF†	+0.346	0.100
Angiostatic		
CXCL9	+0.014	0.722
CXCL10	+0.137	0.618
CXCL11	−1.350	0.027
IFN-γ**^‡^**	−3.858	0.013
Clinical factors		
Hypertension	−2.275	0.006
Diabetes	−1.017	0.053
Current tobacco use	−0.418	0.431
Family history of coronary artery disease	−0.214	0.666
Hyperlipidemia	+2.087	0.032
Prior angina pectoris	−0.566	0.259
Peripheral vascular disease	+0.088	0.891
Congestive heart failure	−0.522	0.586
History of cardiac arrhythmias	−0.200	0.797
Prior myocardial infarction	+0.727	0.165
Prior stroke	−1.203	0.187
Prior percutaneous coronary intervention	−0.841	0.120
Prior coronary artery bypass surgery	−1.734	0.006
Presence of an occluded artery	+4.302	<0.0001
Number of diseased vessels	−0.003	0.990

*VEGF =  vascular endothelial growth factor, †bFGF =  basic fibroblast growth factor, **^‡^**IFN =  interferon.

**Table 3:** Best fit (multivariate) model for the presence of coronary artery collaterals.

Variable	Estimate (x10^4^)	*p* value
CXCL5	+0.609	0.018
CXCL8	+2.555	0.031
CXCL11	−2.409	0.002
CXCL12	+1.389	0.033
IFN-gamma	−3.833	0.021
Hyperlipidemia	+1.924	0.047
Hypertension	−2.431	0.007
Diabetes	−1.729	0.004
Presence of an occluded artery	+4.588	<0.0001

IFN = interferon.

### Determinants of the extent of collaterals

**Figure 1:**
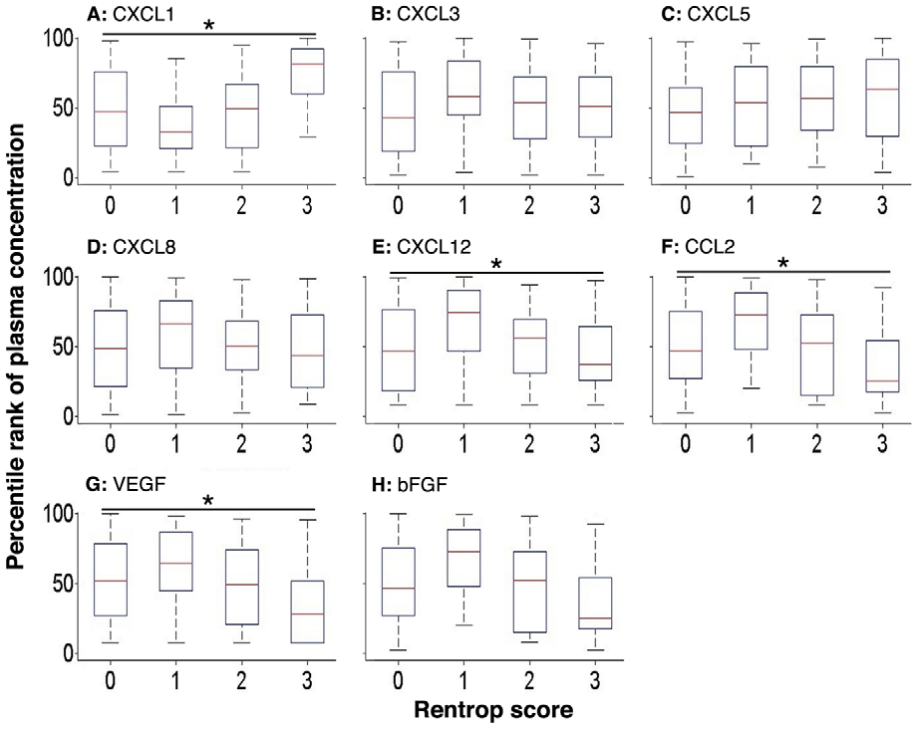
Box plots (median and interquartile range) of percentile rank of plasma concentrations of cytokines that promote vascular remodeling: CXCL1 (panel A), CXCL3 (panel B), CXCL5 (panel C), CXCL8 (panel D), CXCL12 (panel E), CCL2 (panel F), VEGF (panel G), and bFGF (panel H). CXCL1 (*p<0.0001), CXCL12 (*p = 0.0022), CCL2 (*p = 0.0002), and VEGF (*p = 0.008) significantly correlated across increasing Rentrop scores.

**Figure 2:**
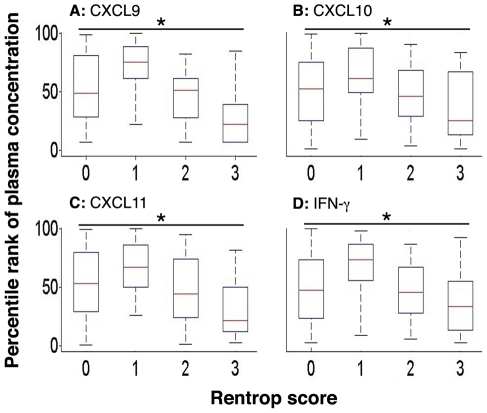
Box plots (median and interquartile range) of percentile rank of plasma concentrations of cytokines that inhibit vascular remodeling: CXCL9 (panel A), CXCL10 (panel B), CXCL11 (panel C), and IFN-γ (panel D). All cytokines significantly correlated across increasing Rentrop scores: CXCL9 (*p<0.0001), CXCL10 (*p = 0.002), CXCL11 (*p = 0.0002), and IFN-γ (*p = 0.0004).

In order to determine whether chemokine levels are predictive of the *extent* of collateralization, when present, we measured plasma levels of the individual cytokines in patients with collaterals (Rentrop score of 1 to 3) and without collaterals (Rentrop score of 0) (Table 4, *p* values represent differences among groups, one-way ANOVA). Increasing Rentrop scores (representing more extensive collateralization), were associated with increasing median plasma levels of the angiogenic CXC chemokine, CXCL1 (*p*<0.0001 for differences among groups, one-way ANOVA) (Table 4, Figure 1). Increasing Rentrop scores were also associated with progressively lower levels of the angiostatic CXC chemokines, CXCL9 (*p*<0.0001), CXCL10 (*p* = 0.002), and CXCL11 (*p* = 0.0002). IFN-γ is a potent inducer of the angiostatic chemokines CXCL9, CXCL10, and CXCL11, and the levels of IFN-γ were similarly negatively correlated to the extent of coronary collaterals (*p* = 0.0004) (Table 4, Figure 2). In addition, we found that CXCL12 (*p* = 0.022), VEGF (*p* = 0.008) and CCL2 (*p* = 0.0002) had a significant negative correlation across increasing Rentrop scores (Table 4, Figure 2). There was no difference in the levels of bFGF between patients with and without collaterals (Table 4, Figure 2).

**Table 4:** Plasma levels of angiogenic and angiostatic factors according to collateralization.

	Rentrop 0(n = 68)		Rentrop 1(n = 28)		Rentrop 2(n = 34)		Rentrop 3(n = 26)		p value
	Medianpg/ml	IQR^§^	Medianpg/ml	IQR^§^	Medianpg/ml	IQR^§^	Medianpg/ml	IQR^§^	
Angiogenic									
CXCL1	6358	1674–0017	2798	1386–7429	6702	1276–15147	24966	10206–138085	<0.0001
CXCL3	667	235–1503	1045	679–1950	960	345–1394	876	381–1384	0.278
CXCL5	6542	4538–9431	7821	4251–13609	8517	5058–14425	9247	5081–21997	0.272
CXCL8	1120	468–2722	2095	759–3642	1190	679–2212	1024	420–2845	0.398
CXCL12	2234	1888–10176	9989	2234–11274	2353	2043–9640	2107	1991–3099	0.022
CCL2	80	40–168	152	83–486	90	22–156	39	27–95	0.0002
VEGF*	1565	660–2579	1947	1266–3710	1428	262–2450	761	0–1700	0.008
bFGF†	0	0–1170	0	0–10990	0	0–4177	52	0–4820	0.555
Angiostatic									
CXCL9	4905	1005–22397	17381	8951–37874	5394	848–9150	392	0–3019	<0.0001
CXCL10	1375	737–2251	1664	1324–3692	1232	795–1967	722	580–2008	0.002
CXCL11	6085	3568–9169	7710	5664–11733	5349	3252–8558	3155	2455–6011	0.0002
IFN-γ^‡^	311	147–565	552	371–1204	290	154–512	189	92–431	0.0004

*VEGF =  vascular endothelial growth factor, †bFGF =  basic fibroblast growth factor, **^‡^**IFN =  interferon, ^§^IQR = inter-quartile range.

## Discussion

In patients with chronic IHD, coronary collateralization maintains myocardial viability in the collateral-fed distribution [27] and is associated with fewer and smaller myocardial infarctions [5], less ventricular aneurysm formation, better left ventricular function, less arrhythmias and better survival compared to those who do not recruit collaterals [2], [12], [13], [14], [28], [29]. It is recognized that patients with similar extent and severity of coronary artery disease exhibit marked heterogeneity in the presence and extent of angiographically detectable spontaneous coronary collaterals; this heterogeneity is not explained by traditional cardiac risk factors. Our multivariable model, which includes clinical factors and cytokine levels, suggests that the angiogenic and angiostatic CXC chemokines play an important role in both the presence and extent of spontaneously visible coronary collaterals.

Prior studies that examined the role of clinical factors on coronary collateralization have yielded conflicting results: For example, in some studies, diabetes mellitus was found to be a negative predictor of the presence of collaterals [30], [31], [32], [33], [34], while in others it was not [35], [36], [37], [38], [39]. In the present study, we found a negative association between diabetes mellitus and coronary collateralization and a positive association with hyperlipidemia, but independent of HMG-CoA reductase inhibitor therapy as has been reported by others [40], [41]. As expected, we also found that patients who had at least one occluded coronary artery were more likely to have collaterals than those who did not.

Substantial preclinical data have implicated growth factors as critical mediators of collateral formation in animal models of hind limb ischemia (reviewed in [42]). Based on this literature, studies of mediators of vascular remodeling in humans have primarily focused on the role of the growth factors VEGF and bFGF in coronary collateralization in chronic IHD [17], [18], [19], [20], [21], [34], [43], [44]. With regard to the role of growth factors as predictors of coronary collaterals, studies have been inconsistent, with some [18], [21] but not others [17], [19], [20] reporting a positive association between plasma growth factors and the presence of collaterals. For instance, in a study with a similar design to ours, plasma levels of VEGF, PDGF, bFGF, or hepatocyte growth factor did not differ significantly between patients with and without coronary collaterals [20]. Surprisingly, we found that VEGF and bFGF levels were not associated with the presence of collaterals; moreover, we found a *negative* association between plasma VEGF concentration and the extent of collateralization when present.

The role of chemokines in mediating vascular remodeling has been extensively documented in diverse biological settings, with the notable exception of revascularization of ischemic myocardium. In one study using a canine model of ischemia/reperfusion injury, investigators showed that short periods of ischemia/reperfusion that were not sufficient to produce a myocardial infarction resulted in increased expression of CCL2 mRNA in the ischemic myocardium suggesting a potential role in angiogenesis [45]. Investigators have reported the over-expression of CXCL8 in atherectomy samples from human coronary artery plaque [46]. Others have shown myocardial chemokine and chemokine receptor expression in human end-stage heart failure [47], but their specific contribution to vascular remodeling has not been established. While these studies primarily concentrated on specific chemokines such as CCL2 and CXCL8, we present data on a complete panel of angiogenic and angiostatic chemokines. Lastly, in a previous clinical study, a predictive model incorporating plasma chemokine levels identified individuals with clinically significant coronary artery disease with better resolution than traditional cardiac risk factors [48]. However, this study did not address the issue of collaterals and the model did not include angiogenic CXC chemokine ligands.

Interestingly, angiogenic and angiostatic factors associated with the *presence* or *absence* of collaterals were not found to be necessarily associated with the *extent* of collateralization in our dataset: for example, CXCL5, CXCL8, CXCL11, CXCL12, and IFN-γ added significant independent information about the presence or absence of collaterals (Table 3), while CXCL1, CCL2, CXCL9, and CXCL10 added information regarding the extent of collateralization (Table 4, Figures 1 and 2). We interpret this finding as follows: local myocardial ischemia may serve as a stimulus for angiogenic chemokine secretion and collateral recruitment. As the collateral is successfully recruited, the ischemic stimulus is no longer operational, resulting in downregulation of the angiogenic ligand. Our model suggests that while arterial occlusion, but not extent of coronary artery disease, is an important first step in recruiting collaterals, their subsequent development is affected by circulating chemokine levels.

### Limitations

Our study has several limitations. First, we may have underestimated the presence of collaterals by measuring only spontaneously visible coronary collaterals [14], [15], [18], [28], [29]. Second, due to the relatively small number of patients, our study may be under-powered to detect significant differences in baseline demographics between the two groups. Third, it is possible that there were differences in factors we did not collect, including the chronicity of angina and participation in a regular exercise program. Fourth, the measurement of CXC chemokines in plasma may be subject to methodological and biological variability. However, we minimized such variability by collecting the samples before the first administration of heparin, and by rapidly cooling and processing the samples. The plasma samples were frozen at −80°C in multiple aliquots from each patient, and all measurements were taken on samples that had been thawed only once. Biological variability was also minimized since all our patients were fasting and at steady-state prior to angiography. Although we did not enroll patients with known unstable angina or myocardial infarction, it is possible that recent symptoms in the peri-catheterization period that were not captured on our history could have affected our results. Nonetheless, since the plasma half-lives of chemokine ligands are short (∼2 hrs), plasma levels are more likely representative of on-going myocardial ischemia than remote events. Finally and most importantly, our results reflect an association but do not establish a causal relationship between the presence and extent of coronary artery collaterals and levels of circulating chemokines, which is not possible in a human system.

The biological basis for the heterogeneity in coronary collateralization in patients with chronic IHD has not been established and is important to patient outcomes. Our findings indicate that an occluded coronary artery is highly correlated with the presence of a spontaneously visible collateral, and plasma concentrations of angiogenic and angiostatic chemokines (but not growth factors) add significant independent information regarding the presence and extent of collaterals, supporting the hypothesis that these molecules are associated with recruitment of coronary collaterals to the chronically ischemic myocardium.

The present work has two potential implications: first, it provides insight into a plausible biological mechanism that underlies the clinically observed heterogeneity in the degree of spontaneously visible coronary collateralization in patients with chronic IHD. If future studies identify angiogenic and angiostatic chemokines as mediating collateral recruitment in this population, this may translate into potential therapeutic options. Second, the present study shows that a statistical model can identify the presence and extent of spontaneous collaterals in a cross-sectional analysis. If this model is found to identify IHD patients destined to recruit collaterals in future longitudinal studies, it may impact the management of this common illness. Lastly, the mechanism(s) behind the selective increase in angiogenic chemokines accompanied by a reduction in IFN-gamma inducible angiostatic CXC chemokines seen in our study is unknown and warrants further study including the possibility of genetic variation.
